# A Variant In the Abo Gene Explains the Variation in Soluble E-Selectin Levels—Results from Dense Genotyping in Two Independent Populations

**DOI:** 10.1371/journal.pone.0051441

**Published:** 2012-12-28

**Authors:** Mahir Karakas, Jens Baumert, Marcus E. Kleber, Barbara Thorand, Dhayana Dallmeier, Günther Silbernagel, Tanja B. Grammer, Wolfgang Rottbauer, Christa Meisinger, Thomas Illig, Winfried März, Wolfgang Koenig

**Affiliations:** 1 Department of Internal Medicine II – Cardiology, University of Ulm Medical Centre, Ulm, Germany; 2 Institute of Epidemiology II, Helmholtz Zentrum München, German Research Center for Environmental Health, Neuherberg, Germany; 3 Mannheim Institute of Public Health, Social and Preventive Medicine, Medical Faculty Mannheim, University of Heidelberg, Mannheim, Germany; 4 Division of Endocrinology, Diabetology, Nephrology, Vascular Disease, and Clinical Chemistry, Department of Internal Medicine, Eberhard-Karls-University Tübingen, Tübingen, Germany; 5 Research Unit of Molecular Epidemiology, Helmholtz Zentrum München, German Research Center for Environmental Health, Neuherberg, Germany; 6 Hannover Unified Biobank, Hannover Medical School, Hannover, Germany; 7 Clinical Institute of Medical and Chemical Laboratory Diagnostics, Medical University of Graz, Graz, Austria; 8 Synlab Academy, Synlab Services GmbH, Mannheim, Germany; Johns Hopkins University, United States of America

## Abstract

**Background:**

Elevated soluble (s) E-selectin levels have been associated with various cardiovascular diseases. Recently, genetic variants in the ABO blood group have been related to E-selectin levels in a small cohort of patients with type 1 diabetes. We evaluated whether this association is reproducible in two large samples of Caucasians.

**Methodology/ Principal Findings:**

Data of the present study was drawn from the population-based MONICA/KORA Augsburg study (n = 1,482) and the patients-based LURIC study (n = 1,546). A high-density genotyping array (50K IBC Chip) containing single-nucleotide polymorphisms (SNPs) from E-selectin candidate genes selected on known biology of E-selectin metabolism, mouse genetic studies, and human genetic association studies, was used for genotyping. Linear regression analyses with adjustment for age and sex (and survey in KORA) were applied to assess associations between gene variants and sE-selectin concentrations. A number of 12 SNPs (in KORA) and 13 SNPs (in LURIC), all from the ABO blood group gene, were significantly associated with the log-transformed concentration of E-selectin. The strongest association was observed for rs651007 with a change of log-transformed sE-selectin per one copy of the minor allele of −0.37 ng/ml (p = 1.87×10^−103^) in KORA and −0.35 ng/ml (p = 5.11×10^−84^) in LURIC. Inclusion of rs651007 increased the explained sE-selectin variance by 0.256 in KORA and 0.213 in LURIC. All SNPs had minor allele frequencies above 20% showing a substantial gene variation.

**Conclusions/ Significance:**

Our findings in two independent samples indicate that the genetic variants at the ABO locus affect sE-selectin levels. Since distinct genome-wide association studies linked the ABO gene with myocardial infarction (MI) in the presence of coronary atherosclerosis and with coronary artery disease, these findings may not only enhance our understanding of adhesion molecule biology, but may also provide a focus for several novel research avenues.

## Introduction

Atherosclerosis involves the recruitment and transendothelial migration of inflammatory cells into vessel walls, which is mediated by cellular adhesion molecules [1]. This multistep process of the interaction between leukocytes and endothelial cells leading to T cell migration into vessels is initiated by selectins, which by ligand interactions allow lymphocytes rolling along the endothelial lining, thereby initiating atherosclerosis. E-selectin is a cell adhesion molecule expressed only on endothelial cells activated by cytokines [2]. The local release of the cytokines IL-1 and TNF-α by damaged cells induces the over-expression of E-selectin on endothelial cells of nearby blood vessels. Circulating concentrations of sE-selectin have been associated with cardiometabolic diseases like diabetes, carotid atherosclerosis and coronary heart disease (CHD), and also with the metastatic potential of some cancers including colorectal cancer and recurrences [3], [4]. Since recently genetic variants in the ABO blood group have been related to E-selectin levels in a small cohort of patients with type 1 diabetes [5], we aimed to evaluate whether this association is reproducible in two large samples of Caucasians.

## Methods

### Study population

The MONICA/KORA (MONItoring of trends and determinants in CArdiovascular disease/ Cooperative Health Research in the Region of Augsburg) Augsburg study is a series of population-based surveys conducted in the region of Augsburg in Southern Germany [6], [7]. The data for the present study was drawn from a subcohort randomly selected by sex and survey from surveys S1 to S3 conducted between 1984 and 1995 [8], [9] and named KORA throughout the manuscript. After exclusion of subjects with no information on sE-selectin concentrations or IBC 50K Chip genotype data or with individual call rates <0.95, the study population for KORA consisted of 1,482 participants (811 men, 671 women) aged 35 to 74 years. All participants were from European ancestry.

The Ludwigshafen Risk and Cardiovascular Health (LURIC) study consists of 3,316 Caucasian patients who were referred for coronary angiography to a tertiary care center in southwestern Germany from 1997 to 2000 [10]. After exclusion of subjects with no information on sE-selectin concentrations or on IBC 50K Chip genotype data or with individual call rates <0.95, the study population for LURIC consisted of 1,546 participants (1084 men, 462 women) aged 17 to 92 years.

In both studies, blood samples for phenotype and genotype assessment was ascertained at the same time as well as all other covariates.

### Ethics Statement

The MONICA/KORA study was approved by the local authorities and performed according to the Declaration of Helsinki [11]. For the LURIC study, the “Ärztekammer Rheinland-Pfalz” gave ethical approval. Written informed was obtained from all study participants in the MONICA/KORA and LURIC study.

### Phenotype measurements

In KORA, E-selectin concentrations were measured with commercially available enzyme-linked immunosorbant assays (R&D Systems, Abington, UK). The intra- and inter-assay coefficients of variation of quality control test were 3.3% and 6.3%. In LURIC, sE-selectin concentrations were measured using the Human sE-selectin assay (R&D Systems GmbH, Wiesbaden, Germany) on a Rosys Plato. In both studies, the distribution of sE-selectin concentrations was skewed to the right and therefore (natural) log-transformed to reach an approximately normal distribution. Details regarding sE-selectin measurements for both studies are given in Table S1.

### Genotyping

In both studies, SNPs were genotyped within the HumanCVD Genotyping Kit (IBC 50K Chip) as suggested by the manufacturer (Illumina, Inc., San Diego, USA). The genotyping was carried out using the CVD-Upenn_Phase1 manifest with the Beadstudio software (Genotyping module v.3.2.29, no call threshold 0.15) in KORA, and using the CVD-Upenn_Phase2 manifest in LURIC. SNPs with minor allele frequency <0.01 and SNP call rate <0.98 were excluded in both studies before the analyses leading to an overall number of SNPs of 30,984 in KORA and 35,980 in LURIC. Details of genotyping are given in Table S1.

The blood samples for phenotype and genotype assessment was ascertained at the same time as well as the other covariates.

### Analytical interference analysis

Blood group A plasma was mixed 1∶1 or 1∶2 with a monoclonal anti-A antibody (Ortho-Clinical Diagnostics, Rochester NY), and incubated 10 minutes or 60 minutes at room temperature, or 60 minutes or 12 hours at 4°C before assaying sE-selectin levels by standard technique [12]. To exclude the possibility that the antibody itself interfered with the assay, the same procedure was repeated with plasma from O blood group individuals. Finally, plasma from O group individuals, which contains both anti-A and anti-B polyclonal antibodies, was mixed with plasma from A group individuals in 1∶1 ratio, again with incubation as above and measurement of sE-selectin concentrations.

### Statistical analysis

In both studies, a linear regression with adjustment for age and sex (and additionally for survey in KORA) was performed using log-transformed sE-selectin as a continuous trait and assuming an additive genetic model. Moreover, a multiple linear regression with additional adjustment for BMI, smokers, actual hypertension, history of CHD, history of diabetes, total cholesterol and HDL-C was performed in both studies. Furthermore, a conditional analysis was performed by additionally adjusting for potential top regions. For SNPs on chromosome 23, adjustments were made without sex.

The association analyses were done by the genetic-statistical software PLINK (http://pngu.mgh.harvard.edu/purcell/plink) [13].

The significance threshold was set to 2.4×10^−6^ to account for multiple testing based on about 20,500 independent tests after accounting for linkage disequilibrium (pruned dataset) with assuming a false-positive rate of 5% and be has been used in other studies utilizing the same genotyping array [14], [15].

## Results

### Study description

Overall, about 3,000 participants were included in the present analysis: 1,482 from KORA and 1,546 from LURIC. The distribution of basic characteristics for both studies is shown in Table 1. Subjects from LURIC were older, with a mean age of 62.5 years (standard deviation 10.9) compared to subjects from KORA (mean age 52.5 years ±10.5) and were more frequently males (70.1% versus 54.7%). The median sE-selectin concentration was lower in LURIC than in KORA (34.0 ng/ml ±22.1 versus 53.7 ng/ml ±29.1).

**Table 1:** Description of the study population.

	KORA	LURIC
Number of participants	1,482	1,546
Age (years)	52.5 (10.5)	62.5 (10.9)
Male sex (%)	54.7	70.1
BMI (kg/m^2^)	27.2 (4.0)*	27.2 (3.9)
Smokers (%)	25.4	19.0
Systolic blood pressure (mmHg)	134.4 (19.4)	141.9 (23.6)
Diastolic blood pressure (mmHg)	81.7 (11.2)	80.7 (11.3)
Hypertension (%)	42.9	72.5
History of CHD (%)	2.4	78.8
History of diabetes (%)	5.5	37.8
Total cholesterol (mg/dl)	237.9 (44.4)	192.6 (37.9)
HDL-C (mg/dl)	56.0 (16.7)	38.6 (10.7)
Lipid-lowering medication (%)	3.58	46.6
sE-selectin (ng/ml)**	53.7 (39.2–69.3)	34.0 (24.5–44.4)
Log (sE-selectin) (ng/ml)	3.96 (0.44)	3.50 (0.46)

Values denote %, in case of continuous variable, mean (standard deviation) is given.

* only n = 1,477 subjects; ** Median (lower-upper quartile).

### Association analyses

**Figure 1:**
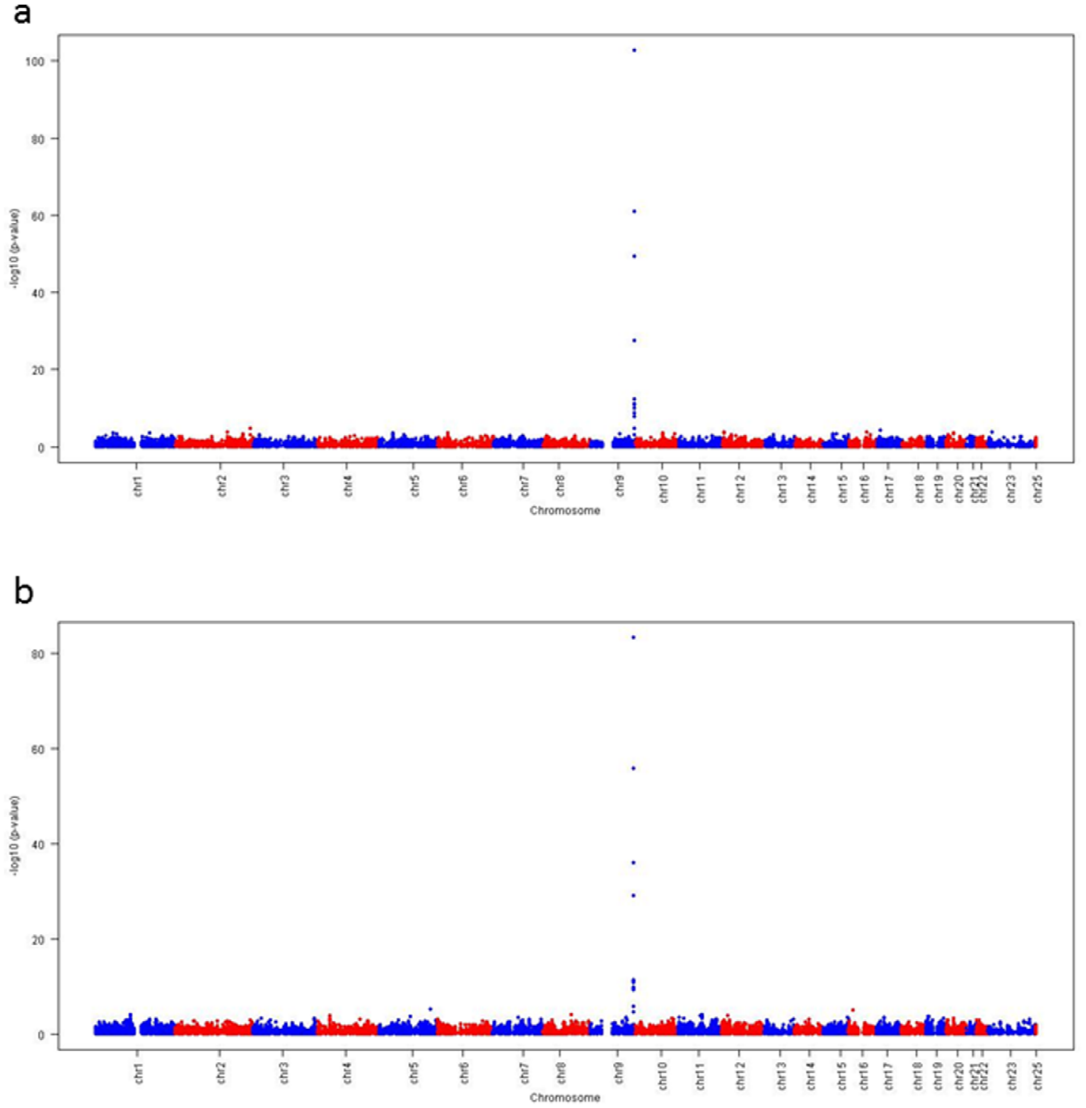
Manhattan plots for KORA (a) and LURIC (b).

**Figure 2:**
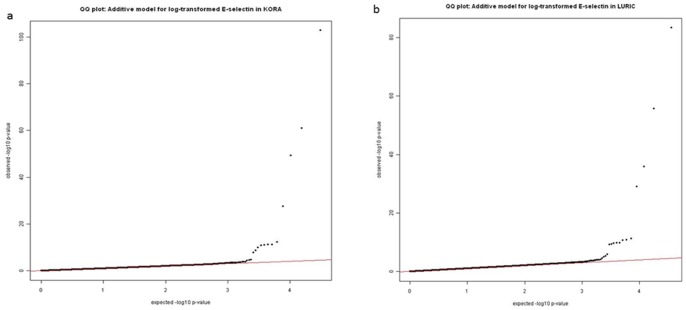
Q-Q plots for KORA (a) and LURIC (b).

The associations between IBC 50K Chip array SNPs and sE-selectin levels are displayed as Manhattan plots in Figure 1a (for KORA) and 1 b (for LURIC) by showing the p values for each chromosome and position on a log-scale. The respective Q–Q plots are shown in Figure 2). The genomic inflation factor was 1.10 in KORA, and 1.06 in LURIC.

These age and sex-adjusted association analyses revealed 12 SNPs in KORA and 13 SNPs in LURIC which had p values below the significance threshold of 5×10^−6^ and were in both studies all located in the ABO blood group gene (ABO) on chromosome 9. All SNPs had minor allele frequencies above 20% showing a substantial gene variation. An additional adjustment for BMI, smokers, actual hypertension, history of CHD, history of diabetes, total cholesterol and HDL-C revealed very similar beta estimates and p values for rs651007 (KORA: −0.364 (SE 0.015) and p value 3.64e-108, LURIC: −0.353 (SE 0.016) and p value 4.72e-93) as in the age- and sex-adjusted regression analyses. As shown in Table 2, the strongest association was observed in both studies for rs651007, with a change of log-transformed E-selectin concentration per one copy of the minor allele of −0.37 ng/ml (p = 1.87×10^−103^) in KORA and −0.35 ng/ml (p = 5.11×10^−84^) in LURIC. In both studies, the model fit assessed by the R^2^ increased strongly from the model without rs651007 to the model with rs651007: from 0.044 to 0.300 for KORA, and from 0.021 to 0.234 for LURIC meaning R^2^ increases of 0.256 and 0.213.

**Table 2:** Association of the top SNP rs651007 with log-transformed sE-selectin, adjusted for age and sex (and additionally for survey in KORA).

	KORA	LURIC
Number of participants	1,479*	1,543*
SNP	rs651007	rs651007
Position (Locus)	135143696 (ABO)	135143696 (ABO)
Chromosome	9	9
Alleles major > minor	G>A	G>A
Minor allele frequency	22.1%	26.0%
Beta estimation (standard error)	−0.373 (0.016)	−0.347 (0.017)
P value	1.87e-103	5.11e-084
Change in R^2^ **	0.256	0.213

*Coding allele was the minor allele.*

* *For three subjects in KORA and in LURIC, information on rs651007 was missing.*

** *Change in R^2^ of age- and sex-adjusted models (+ survey in KORA) with and without rs651007.*

The multiple-adjusted regression analyses revealed very similar associations for rs651007 in both studies, as in the age- and sex-adjusted regression analyses (KORA: −0.36, p = 3.64×10^−108^, LURIC: −0.35, p = 4.72×10^–93^).

To assess whether other SNPs might be significantly associated with log-transformed sE-selectin concentrations independently of rs651007, we conducted a conditional analysis, i.e. an analysis additionally adjusting for rs651007. These analyses found no other associated SNP in both studies, as all SNPs were not significantly associated with log-transformed E-selectin concentrations: the beta estimates attenuated substantially for all SNPs with p values between 0.0002 and 0.775 in KORA and between 0.00006 and 0.593 in LURIC.

### Analytical interference analysis

Although E-selectin is not known to bear the ABO histo-blood group antigen, this possibility could not be ruled out. We therefore excluded the possibility that the association between A histo-blood group antigen and lower sE-selectin was the consequence of a lower affinity of the antibodies used in the sE-selectin assay for E-selectin carrying the A antigen. We thus hypothesized that blocking the A antigen sites with either polyclonal or monoclonal antibodies would result in lower sE-selectin values if E-selectin does indeed carry ABO histo-blood group antigen and if the A antigen is located in the proximity of one of the two antibody binding sites used by the immunoassay. No differential effects of the mixing procedures were observed suggesting that the A blood group antigen was not interfering with measurement of sE-selectin levels (data not shown).

## Discussion

In these analyses we could confirm previous much smaller studies that were mainly conducted in cohorts with diabetes, and extend this association to healthy subjects and patients with CHD [5]. Using dense-genotyping in two large independent populations, our findings indicate that sE-selectin concentrations are mainly determined by genetic variants at the ABO locus. Regarding the top SNP, sE-selectin concentrations decreased in both studies per one copy of the minor allele. In both studies, the model fit assessed by the R^2^ increased strongly after addition of rs651007, and conditional analysis revealed that remaining SNPs are not independently associated with sE-selectin levels, indicating that variance in sE-selectin concentrations were mainly explained by rs651007 only. We tested the interaction of rs651007 and diabetes on E-selectin and found no modifications of the associations seen by p values for interaction of 0.52 in KORA and 0.49 in LURIC. Therefore, it can be concluded that the association between rs651007 and sE-selectin was not modified by diabetic condition. Like all SNPs found to be significantly associated with variation in sE-selectin levels in our study, the top SNP rs651007 is located within the ABO gene, which encodes a glycosyltransferase that transfers sugar residues to the H antigen and thereby determines the ABO blood group [16]. The main SNP rs651007 is located upstream. The eQTL – SNP-Gene analysis showed no effect of rs651007 on ABO expression. It has been shown that the A1 subtype, which is perfectly tagged by the minor allele of SNP rs651007, has a >30-fold higher transferase activity than the A2 subtype, thereby implicating that the association found may represent an effect of the ABO group A1 subtype [17]. It has been hypothesized that the increased glycosyltransferase activity in individuals carrying the A1 allele might have an effect on the production and clearance of adhesion molecules, thereby influencing their levels in circulation [18]. Furthermore, studies from the field of microbiology indicated that leukocytes interact with ABO antigens on endothelium, and that the A antigen promotes stronger binding of leukocytes to selectins on the vascular wall, thereby protecting it from enzymatic cleavage and leading to decreased selectin concentrations in serum and elevated concentrations on endothelial cells [19]. This might explain the findings of multiple studies linking the A allele to increased risk of MI, vascular disease and venous thromboembolism [20]. In accordance, recently distinct genome-wide association studies linked the ABO gene with MI in the presence of coronary atherosclerosis, with coronary artery disease, with type 2 diabetes and with venous thromboembolism [21]–[24]. Furthermore, our top SNP tags rs507666, which was the top SNP in GWAS of circulating concentrations of LDL-cholesterol and ICAM-1 [25], [26]. In this context, the association of several published loci for atherosclerotic diseases might be mediated by diverse processes that promote inflammation and atherogenesis, and it appears that a substantial subset of risk loci have pleiotropic effects.

Although we excluded the possibility of an analytical interference to explain the association, the exact mechanism linking histo-blood group antigen to lower E-selectin concentrations, especially in the context of several candidate biomarker reports that linked lower sE-selectin concentrations with lower incidence of cardiovascular diseases, remains elusive. Among the different hypotheses, it remains possible that sE-selectin bears the A antigen, a modification that might increase its clearance by increasing its affinity for its receptors and/or decrease its secretion from membrane-bound E-selectin. But it is also conceivable that elevated E-selectin levels in high-risk patients represent an epiphenomenon of vessel wall pathology, or a counter-regulatory per se protective mechanism. This is supported by the finding, that in the KORA study population, i.e. apparently healthy, middle-aged subjects, median sE-selectin concentrations were clearly higher than in the LURIC population, i.e. subjects referred to coronary angiography (53.7 ng/ml versus 34.0 ng/ml). Further studies investigating the association between ABO variants and cardiovascular disease, as well as perhaps Mendelian randomization may determine the potentially causal nature of sE-selectin in cardiovascular diseases and provide a more comprehensive assessment of the biological and clinical relevance of the association.

These findings may not only enhance our understanding of adhesion molecule biology, but may also provide a target for several novel research avenues. The increased power to identify variants of small effects, afforded by large sample size and the dense genetic coverage including low-frequency SNPs within loci of interest, has resulted in the identification of associations between previously unreported genes and sE-selectin concentrations, which implicate a regulatory role of histo-blood group antigens in inflammatory adhesion processes.

## Supporting Information

Table S1 **Study information.**
(DOC)
