# Supplementary material for: A Variant In the Abo Gene Explains the Variation in Soluble E-Selectin Levels—Results from Dense Genotyping in Two Independent Populations
Source: PLoS One. 2012 Dec 28;7(12):e51441. doi: 10.1371/journal.pone.0051441 (PMC3532506; doi:10.1371/journal.pone.0051441)
Supplement: Table S1 — Study information. (DOC) [file pone.0051441.s001.doc]

**Supplementary Table 1:** Study information

|  | KORA | LURIC |
| --- | --- | --- |
| Study population and design: |  |  |
| Ethnicity | Caucasian | Caucasian |
| Study area | Augsburg region, Germany | Rhein-Neckar area, Germany |
| Study type | Population-based | Patient-based |
| E-selectin measurement: |  |  |
| Assay | Immunosorbant assay | Immunosorbant assay |
| Platform | R&D Systems | R&D Systems |
| Genotyping: |  |  |
| Genotyping platform & SNP panel | Illumina, IBCv1 | Illumina IBCv2 |
| Genotyping centre | Helmholtz Zentrum München | Sanger |
| Genotyping calling algorithm | Beadstudio (module v.3.2.29) | Beadstudio (module v.3.2.29) |
| Exclusions: |  |  |
| Individual call rate | < 0.95 | < 0.95 |
| SNP call rate | < 0.98 | < 0.98 |
| Minor allele frequency | < 0.01 | < 0.01 |
| HWE p value | < 1.0x10^-7^ | < 1.0x10^-7^ |
|  |  |  |
| Number of participants after exclusions: | 1,482 | 1,546 |
| Number of SNPs after exclusions: | 30,833 | 35,980 |
| Statistical analyses: |  |  |
| Analysis method | Linear regression | Linear regression |
| Adjustments | Age, sex and survey | Age, sex |
| Software for analysis | PLINK | PLINK |
| Number of SNPs with valid estimations: | 30,257 | 35,334 |
| Genomic Inflation factor (λ) | 1.10 | 1.06 |
